# Phlebitis after Venesection

**Published:** 1829-07-01

**Authors:** 


### PHLEBITIS AFTER VENESECTION.

An interesting case of this description has been related by Mr. Chinnock, a very intelligent surgeon of Brompton, in the March number of the Med. and Phys. Journal. The issue, as unfortunately it almost always is in acute phlebitis, was fatal.

The patient was a labouring man of robust plethoric habit, who was bled at St. George's Hospital in consequence of a fall, and, two or three days afterwards, consulted Mr. Chinnock, with some slight febrile symptoms, which assumed no appearance of severity till the evening of the second day from Mr. C.'s visit. It looked, and was treated like a case of common inflammatory fever, and Mr. Chinnock bled the patient freely from the left arm. Next day, on proceeding to repeat the venesection from the right (the one he had been bled in at the hospital, and of which he had not hitherto complained) the lancet wound was found to be inflamed, the arm and shoulder generally swollen as high as the axilla, and considerable stiffness and pain experienced on moving or meddling with the limb. Twenty leeches were applied to the part, the patient bled again from the other arm, and salines with antimonials, calomel and opium, and a brisk aperient next morning prescribed. On the 4th day he felt himself relieved, but the countenance was anxious — the pulse 120, sharp, and small—the coating of the tongue darker coloured—the tumefaction and sensibility in the neighbourhood of the arm-pit evidently increased. Leeches, poultice, tincture of hyosciamus and acetate of ammonia in camphor mixture, and the opiate at bed-time, were the means employed, but in the night he had two distinct rigors, and on the 5th day was worse. The swelling on that day being greater about the axilla, the pain even agonizing, and the general characters such as to indicate the formation of matter in the axillary region, Mr. Chinnock made a free incision with a sharp-pointed bistoury, and let out a great quantity of healthy pus. Relief, and that very marked, ensued, but it proved to be fleeting and fallacious, for next day there was found to be additional excitement—acute shooting pains were felt running down from the shoulder to the fore-arm—the limb was smaller, but a reddened inflammatory line marked the whole course of the cephalic vein, which was felt distinctly hard and corded, whilst pain was produced in it by pressure, and a little pus made to issue from the orifice—the spirits of the patient were sunk, and with difficulty he agreed to the application of eighteen leeches to the vein, poultice and fomentation, and salines with henbane and opiates. In the evening he again appeared somewhat better, but on the 7th day the typhoid symptoms were unequivocal, and the patient was at times incoherent. The discharge from the wound and from the vein was sanious and thin, the integuments in the axilla flabby and livid, the red line of inflammation of the vein had disappeared, a difficulty was felt in the right side of the chest in drawing in his breath, which could only be imperfectly done, in consequence, not of pain or cough, but a sense of choking. A slight tumefaction, with tenderness and increase of heat, were also perceived in the opposite axilla. From this time he rapidly sank, became delirious, and died in the evening of the 9th day of Mr. Chinnock's attendance upon him. Previous to his death the tumefaction in the left axilla increased, he would not submit to an incision, and the part, like opposite limb, assumed a disposition to gangrene.

*Sectio Cadaveris.* The orifice at the bend of the arm opened distinctly into the vein, which was pervious to air from a blow-pipe. The soft parts immediately around the vein were considerably thickened and required much care to be separated from the vessel. The median cephalic and cephalic were double their natural size, thickened in their coats, the external injected and more vascular than natural, and the vessels altogether looking like an artery rather than a vein. The internal tunic was red and much thickened, and no blood was contained in the cavity of the vessel, which was partially filled with purulent matter in patches like clotted cream. These appearances were observed till within about an inch of the termination of the vein in the axillary, where they ceased; the median basilic and basilic shewed feeble traces of inflammatory action. About an inch *below* the wound the morbid appearances ceased, but dark-coloured semi-fluid matter was formed in the interstices of all the muscles in the front of the arm, whilst the fibres of those which bound the axilla were gangrenous, and neighbouring cellular membrane was sloughy, and injected with sanious matter.

In the left axilla was an ounce and a half of thin grumous fluid, and in the right cavity of the chest, Mr. C. was surprised to discover from four to six ounces of semi-purulent matter. The pleura on that side had contracted some trifling adhesions, but presented no further evidence of inflammation. The right lung was greatly engorged — the opposite side of the chest was healthy—the heart and pericardium sound—the brain and abdominal viscera not examined.

The above was an interesting and well marked case of phlebitis, terminating, as the majority of such cases do, at any rate, of those which we have witnessed, by a disposition to purulent effusions either into the pleural cavity or elsewhere. Mr. Chinnock brings it forward as serving to confirm Mr. Arnott's views, but to this we shall merely refer for the present, as we purpose to defer expressing our opinions on the subject, till the paper of the gentleman in question comes properly under our cognizance.

